# Biocontrol of *Fusarium equiseti* using chitosan nanoparticles combined with *Trichoderma longibrachiatum* and *Penicillium polonicum*

**DOI:** 10.1186/s40694-023-00151-4

**Published:** 2023-02-06

**Authors:** EL-Sayed M. El-Morsy, Yomna S. Elmalahy, Mohamed M. A. Mousa

**Affiliations:** Botany and Microbiology Department, Faculty of Science, Damietta University, New Damietta, 34517 Egypt

**Keywords:** *Fusarium equiseti*, Chitosan nanoparticles, Antifungal, Pathogenic fungi, Antagonistic fungi

## Abstract

A safe and ecofriendly biocontrol of pathogenic *Fusarium equiseti* was developed based on chitosan nanoparticles (CNPs) combined with* Trichoderma longibrachiatum* and *Penicillium polonicum.* Two strains of* F. equiseti* which were isolated from wilting tomato plant as well as three antagonistic fungi including *Trichoderma longibrachiatum* and two strains of *Penicillium polonicum* were isolated from the surrounding soil. All the isolated pathogenic and antagonistic fungi were identified using genomic DNA sequences. The antifungal activity of the three antagonistic fungi were studied against the two strains of *F. equiseti.* Also, CNPs which were prepared according to the ionic gelation method using sodium tripolyphosphate anions in acetic acid solution were used to enhance the antifungal activity of the three antagonistic fungi. The results exhibit that, combination of *T. longibrachiatum* with CNPs and *P. polonicum* with CNPs achieve high antifungal activity against *F. equiseti* by an inhibition rate equal to 71.05% and 66.7%, respectively.

## Introduction

Phytopathogenic fungi seriously affect large numbers of plants, seeds and damage many important crops all over the world. As a result, many crops were spoiled, which led to a decrease in agriculture, both qualitatively and quantitatively [1, 2]. Among these plant pathogenic fungi, *Fusarium* species are considered one of the most important known soil borne plant pathogens [3–5]. *Fusarium* species are widely distributed in many sources such as air, soil, plants, marine ecosystems, and fresh water [6]. Also, *Fusarium* species have the ability to be alive either as chlamydospores in the remains of the infected plants for about 30 years or in the alternative host roots, and cause high levels of damage for many crops such as tomato, pea, potato, bean, wheat, corn and rice with yield losses up to 30–70% [3, 7]. Among *Fusarium* species, *F. equiseti* causes wilt diseases on various plant hosts such as grafted watermelon, grape, cucumber, tomato, cowpea, bean, potato [8–15]. In addition *F. equiseti* was reported to cause root rot of sugar beets [16]. Also, some strains of *F. equiseti*, can produce mycotoxins such as Zearalenone which they are usually detected in combination with other fusariotoxines, such as trichothecenes and fumonisins. All are dangerous toxins to life being [17].

In this context, effective control of *Fusarium* species especially *F. equiseti* is an essential requirement to maintain a safe environment for both human and animals. Although plant pathogenic fungi can be eliminated with chemical fungicides, excessive use of them has many drawbacks including being harmful to soil, causing deviation from the normal flora and fauna system, resistance to pathogens and pollution of the environment [18]. However, biocontrol techniques provide a safe solution for the problems of chemical fungicides, and there are a few publications that deal with biocontrol of *F. equiseti* with moderate inhibition rates. For instance, *Azotobacter nigricans* was reported as antifungal against some *Fusarium* species including *F. sporotrichioides, F. graminearum, F. poae* and *F. equiseti* with a growth inhibition up to 50% [19]. *Bacillus subtilis *was used for controlling *F. solani, F. equiseti* and *F. oxysporum* with inhibition percentage ranged from 51, 66 and 47% after 5 days, respectively [20]. Also, *Streptomyces bellus* was applied against *F. equiseti* and two strains of *F. fujikuroi* and achieved inhibition percentage 55, 43 and 36%, respectively [16].

During the past few years, metal nanoparticles (MNPs) such as silver [21, 22], copper [23], zincite [24], titania [25], gold [26, 27], nickel, and core–shell Ag-SiO_2_ [28] have been attracting interest to be used as a biocontrol for different pathogenic fungi. The antifungal activity of MNPs were promoted from their shape, size distribution, composition, crystallinity, surface chemistry, and agglomeration of the nanoparticles [29]. Although MNPs generally perform well, they cannot be applied in a wide range because they pollute the environment as well as their toxicity [30–32]. As a result, nano natural polymers gain enormous attraction in controlling the pathogenic fungi that may achieve a safe pathway to overcome the problems of chemical fungicides as well as the toxicity of MNPs. Among these nano-polymers, chitosan nanoparticles (CNPs) have attracted great interest to be used as antifungal for many different pathogenic fungi due to their unique properties such as non-toxicity, low cost, biodegradability, high permeability through biological membranes, and wide antifungal activities against numerous phytopathogenic fungi [32–37].

It is stated that biogenic CNPs from four different fungal sources in combination with *Trichoderma asperellum* was effective in suppressing mycelial growth pathogenic fungi including *Fusarium oxysporum* and* F. graminearum* [38, 39]. Alike biogenic CNPs was inhibit the growth of *Fusarium oxysporum ciceri*, *Pyricularia grisea,* and *Alternaria solani* with the rate of inhibition 87%, 92%, and 72%, respectively [40].

Due to the hazard issue of *F. equiseti* and the good properties of CNPs as well as the few published studies deal with the use of CNPs as antifungal against *F. equiseti,* we aim in this study to isolate *F. equiseti* from wilting tomato plant and inhibit their growth using some antagonistic fungi and CNPs. Furthermore, we aimed to study the antifungal activities of these antagonistic fungi combined with CNPs against *F. equiseti*.

## Materials and methods

### Isolation and purification of pathogen and antagonist

The pathogenic and antagonistic fungi were isolated from the infected vascular tissues of tomato (roots and stem) and the surrounding soil collected from Damietta and Dakahlia governorates, Egypt. Pathogenic isolated from surface sterilized (5% hypochlorite solution) root and stem pieces (0.5–1 cm) on potato dextrose agar (PDA) containing traces of Chloramphenicol for 5 to 7 days at 25 ± 27 °C. For isolation of antagonistic fungi, the surrounding soils were air dried and saved to remove large particles. Then 1 ml from each dilution (10^− 1^ to 10^− 5^), was transferred to PDA plates, incubated for 5 days at 25 ± 27 °C. The growing hyphal tips were picked up and preserved on PDA slopes further studies. [41, 42].

### Synthesis of chitosan nanoparticles

CNPs were prepared according to the ionic gelation method of chitosan with Sodium tripolyphosphate anions in acetic acid solution [32]. The prepared chitosan nanoparticles suspension solution was kept at 4 °C for further analysis and use.

### Biological control

#### Effect of CNPs on mycelium radial growth of *F. equiseti*

A radial hyphal growth bioassay was used to test the antifungal activity of CNPs against both *F. equiseti* st.1 and *F. equiseti* st.2 [43]. The pH of both the stock solutions prepared from CNPs (0.05% w/v) and acetic acid (1%) were adjusted at 5.6 by adding drops of NaOH solution (2 M) followed by sterilizing in autoclave at 121 °C and 1.5 atm. To prepare different concentration of CNPs, various volumes of stock solution of CNPs (0, 2, 4, 6, 8 ml) were mixed with the adjusted acetic acid by different volumes of (8, 6, 4, 2, 0 ml), respectively. Eight milliliters of each CNPs solution were mixed with 15 ml of autoclaved PDA medium and poured in sterilized Petri dish to obtain a final concentration of (0, 0.043, 0.086, 0.129, 0.172 mg/ml) CNPs. From a 7-day old culture of *F. equiseti* st.1 and *F. equiseti* st.2, a mycelial piece of uniform size (diameter, 5.0 mm) were cut by corkborer from the peripheral end and inserted in the center of the test Petri dishes. All Petri dishes were incubated in laboratory condition at 25 °C for 7 days, and daily measurements of radial colony growth were taken until the fastest growing colony approached the plate's edge. All the treatments had three replications, and the experiment was carried out twice. By using Vincent's formula (Eq. 1), the percent inhibition rate of the pathogen's mycelia was calculated by comparing the treatment plates to the control (without CNPs).

1 $$\% Inhibition rate= \frac{{M}_{C}- {M}_{t}}{{M}_{c}} \times 100$$

where M_c_ and M_t_ are the mycelia growth in control and the mycelia growth in treatment, respectively.

#### Antagonistic activities of isolated fungi against *F. equiseti*

Dual culture method was used for testing the antagonistic activities of isolated fungi against *F. equiseti* st.1 and *F. equiseti* st.2 [44]**.** This method was established in sterile petri dish by transferring an agar disc (5 mm in diameter) of 7 days old culture of each strain of *F. equiseti* which was cut by sterilized cork borer and then was placed in PDA media in the edge of petri dish. In the opposite edge of petri dish another same sized agar disk of the antagonistic fungi was placed, then incubated in laboratory condition at 25 ± 27 °C for 5 days. The antagonistic activity was recorded after incubation by calculating the inhibition rate percentage according to Eq. (1).

#### Combination of CNPs and antagonistic fungi against *F. equiseti*

An in vitro study was carried out to determine the efficiency of CNPs combined with the three antagonistic fungi against the two strains of *F. equiseti* [45]. In this experiment firstly, each of the three antagonist fungi was inoculated into PDA medium containing CNPs in a concentration (0.172 mg/ml) which exhibit the highest inhibition for *F. equiseti* st.1 and *F. equiseti* st.2. After complete growth of three antagonistic fungi (7 days old culture), a mycelial disc (5 mm diameter) was cut by corkborer and inoculated into another two plates containing *F. equiseti* st.1 and *F. equiseti* st.2 on one side of the PDA plate, respectively. After observing full growth in the control plates, the radial growth of the pathogens was recorded. The percentage of inhibition of mycelial growth was calculated according to Eq. 1.

#### Characterization techniques

Genomic DNA from pure pathogenic and antagonistic fungal cultures were extracted using ABT DNA mini extraction kit (Applied Biotechnology Co. Ltd, Egypt), according to the manufacturer’s instructions. PCR was performed in Veriti™ 96-Well Thermal Cycler (Applied Biosystems). The products of the amplified PCR were submitted to Solgent Co Ltd (South Korea) for gel purification and sequencing. Fourier transform infrared analyses (FTIR) was recorded by using KBr plates on a JASCO FT/IR-4100 Fourier transform infrared spectrometer. Transmission Electron Microscopy (TEM) image for CNPs was acquired by JEOL JEM–2100 microscopy at an accelerating voltage of 200 kV. Zeta Potential analysis was carried out using Malvern Zetasizer Nano-ZS90, Malvern, UK.

## Results and discussion

### Molecular identification of pathogenic and antagonistic fungi

The results obtain from DNA sequences for both pathogenic and antagonistic fungi were trimmed and assembled in Geneious software (Biomatters). Consequently, the trimmed sequences were identified by search in basic local alignment search tool (BLAST) in GenBank and recorded with accession numbers as shown in Table1.

**Table 1:** Isolation sites and identification of pathogenic and antagonistic fungi

Accession number	Locality	Source	BLASTn result identify (%)	Species
ON533655	Gamasa city	Stem	100	*Fusarium equiseti* st.1
ON533656	New Damietta city	Stem and root	100	*Fusarium equiseti* st.2
ON533657	New Damietta city	Clay soil	100	*Trichoderma longibrachiatum*
ON533654	Gamasa city	Clay soil	100	*Penicillium polonicum* st.1
ON533658	New Damietta city	Sandy soil	100	*Penicillium polonicum* st.2

*St* strain

### Characterization of chitosan nanoparticles

**Fig. 1:**
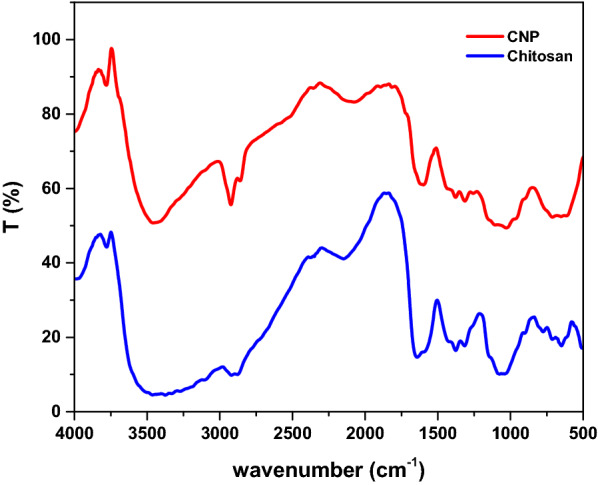
FTIR analysis of chitosan and CNPs

FTIR was used to confirm the formation of CNPs through the interaction between chitosan and TTP. Figure 1 shows FTIR analysis of both chitosan and CNPs. Starting with chitosan, there are two characteristics peaks at 1643 and 900 cm^−1^ which are attributed to amide (-CONH_2_), anhydro glucosidic ring and another peak at 3450 cm^−1^ which is related to primary amine group (NH_2_). Moving into CNPs, the characteristic peaks of amide and primary amine groups are shifted to lower wavenumbers and appear at 1602 and 3425 cm^−1^, respectively. The decrease in stretching frequency could be due to the TPP interaction with ammonium group of chitosan and more hydrogen bonding in chitosan–TPP complex [32].

**Fig. 2:**
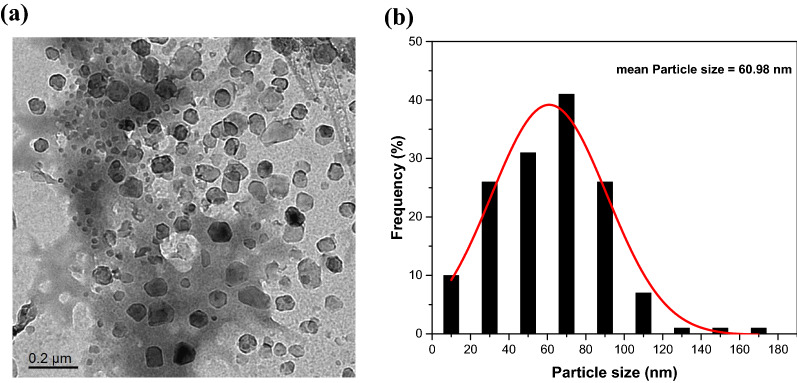
**a** TEM images and **b** particle size distributions of CNPs

Both transmission electron microscopy (TEM) image and particle size distribution of CNPs are presented in Fig. 2. The result shows that CNPs appear as nearly spherical particles with an average particle size equal to 60 nm which is consistent with the results of other papers [7, 32, 37].

**Fig. 3:**
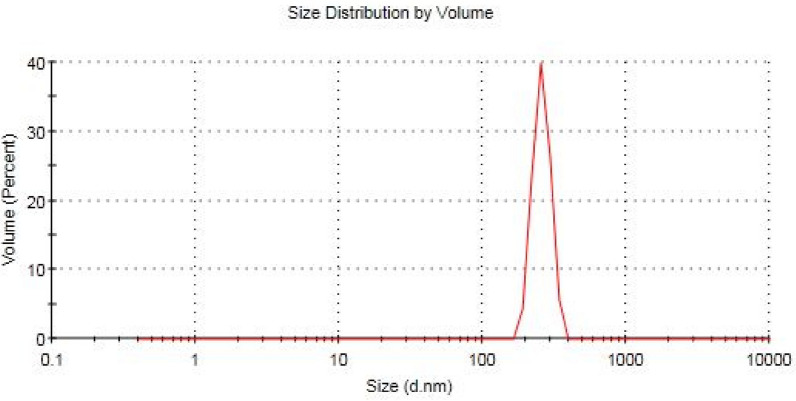
Particle size distribution of CNPs obtained from DLS

Figure 3 shows the particle size distribution of CNPs obtained from dynamic light scattering (DLS). The results illustrate that CNPs have a narrow particle size distribution with a mean average diameter 259.4 nm. Also, particle size of CNPs measured using DLS analysis is much larger than this obtained from TEM. The difference is attributed to the swelling effect of chitosan hydrogel in aqueous solution while a noticeable shrinkage effect appears in the dry solid state at the time of TEM analysis [6]. Furthermore, Zeta potential indicates that CNPs have a positive surface charge with a value of 90.7 mV which support the high stability of CNPs in aqueous solution during this study.

### Biological control

#### Effects of CNPs on mycelium radial growth of *F. equiseti*

**Fig. 4:**
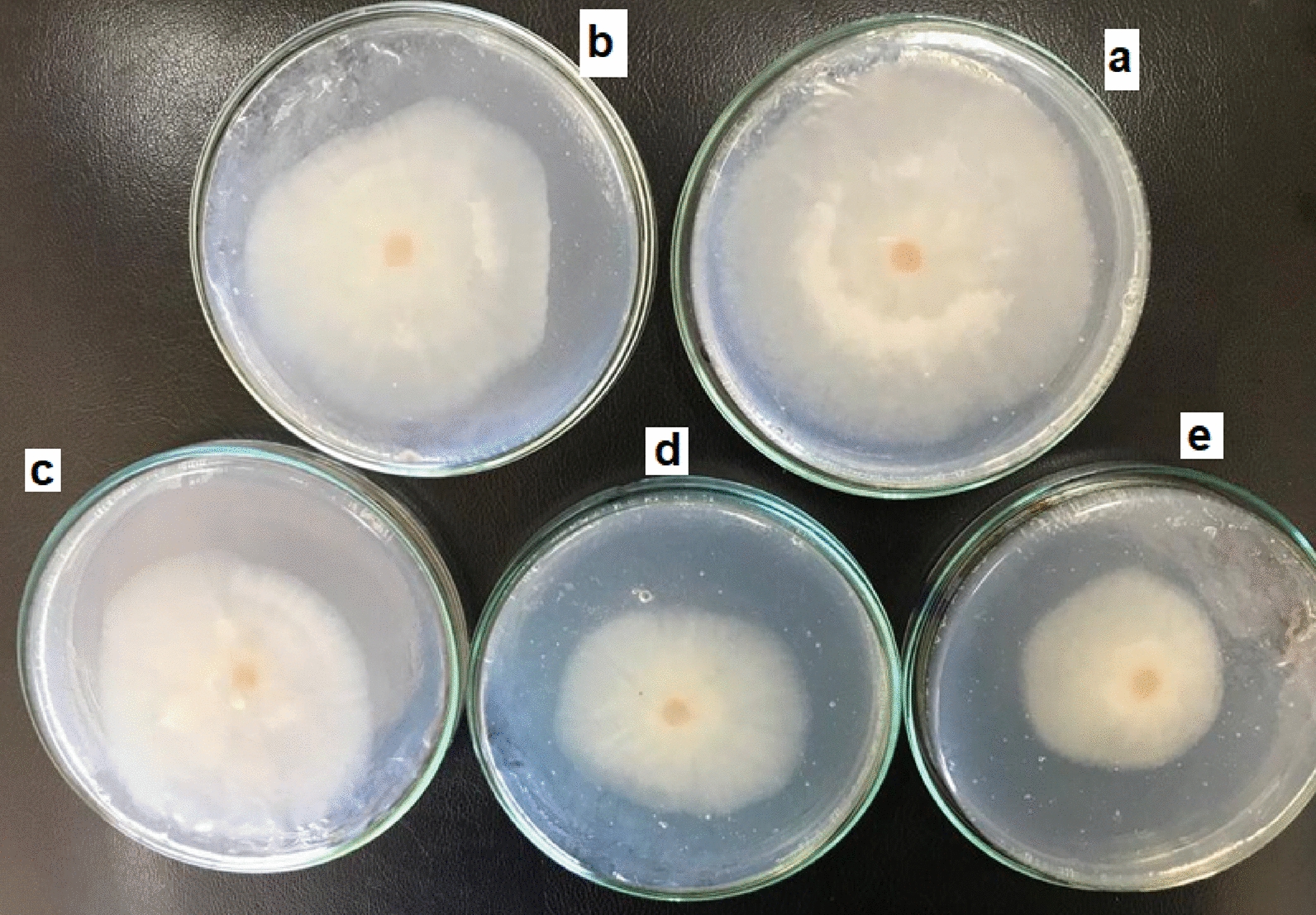
The effect of CNPs on mycelium radial growth of *F. equiseti* st.1 with different concentrations control **a**; 0.043 mg/ml **b**; 0.086 mg/ml **c**; 0.129 mg/ml **d**; 0.172 mg/ml **e** on PDA plate

**Fig. 5:**
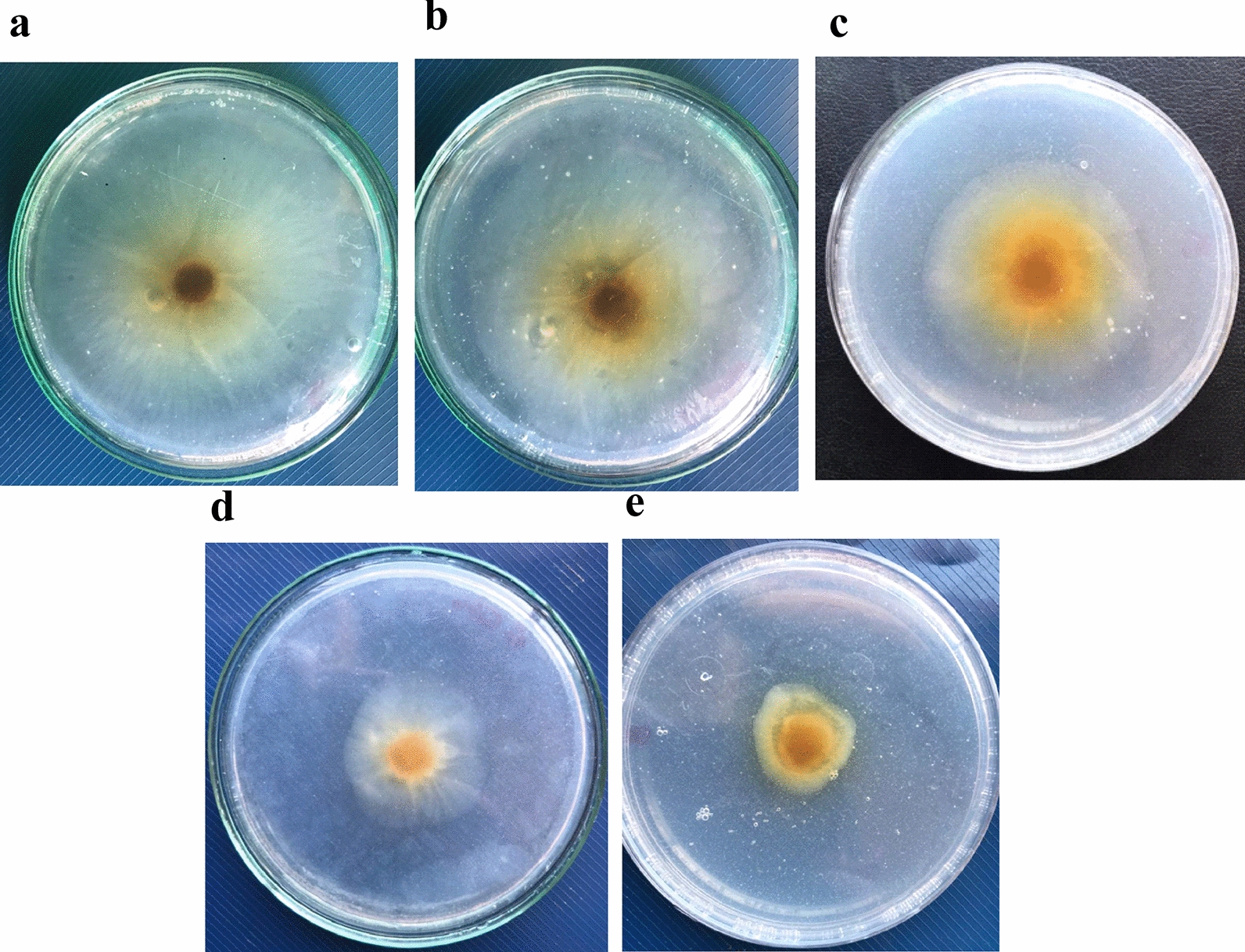
The effect of CNPs on mycelium radial growth of *F. equiseti* st.2 with different concentrations control **a**; 0.043 mg/ml **b**; 0.086 mg/ml **c**; 0.129 mg/ml **d**; 0.172 mg/ml **e** on PDA plate

CNPs are used as a biodegradable polymer for the inhibition of mycelium radial growth of both *F. equiseti* st.1 and *F. equiseti* st.2. The antifungal activity of CNPs appears from the affinity of its cationic amino groups to cellular components [6]. All the experiments were done in PDA media with different concentrations of CNPs from 0.043 to 0.172 mg/ml and incubate at 25 °C for 7 days. Figure 4, 5 and Table 2 present the effect of CNPs on mycelium radial growth of the two strains of *F. equiseti*. The results show that increasing the concentration of CNPs leads to increase the inhibition percentage for both *F. equiseti* st.1 and *F. equiseti* st.2. The maximum inhibition rates for *F. equiseti* st.1 and *F. equiseti* st.2 is found to equal 40.39% and 66% at CNPs concentration 0.172 mg/ml, respectively. While the minimum inhibition rates are 8.81% and 19% at CNPs concentration 0.043 mg/ml for *F. equiseti* st.1 and *F. equiseti* st.2.

**Table 2:** Determination of the effect CNPs on mycelium radial growth of the two strain *F. equiseti*

Treatment by CNPs (mg/ml)	Inhibition rate (%)	
	*F. equiseti* st.1	*F. equiseti* st.2
0.043	8.81	19.34
0.086	15.39	32.59
0.129	29.47	43.72
0.172	40.39	66.00

### Antagonistic activities of isolated fungi against *F. equiseti*

The antagonistic activities are measured by inhibition the growth of the two strains *F. equiseti* which were isolated from wilting tomato plants. The measuring of antagonistic activity used the dual plate method [44]. Table 3 present the effect of *T. longibrachiatum, P. polonicum* st.1 and* P. polonicum* st.2 as antagonistic fungi for inhibition of *F. equiseti* st.1 and *F. equiseti* st.2*.* It is clear from Tables 3, 4 that *T. longibrachiatum* exhibits the higher antifungal activity against *F. equiseti* st.1 and *F. equiseti* st.2 by inhibition rates of 60% and 62.74%, respectively while *P. polonicum* st.1 appears less antifungal activity by inhibition rates of 40% and 51.41% for *F. equiseti* st.1 and *F. equiseti* st.2, respectively. In fact, *Trichoderma* spp. have long history as a biocontrol agent against several pathogenic fusaria such as *Fusarium oxysporum* [46] and Fusarium sudanense [47]. It is also stated that *Tr*.* longibrachiatum* acts as a biocontrol agent of *Fusarium* wilt of cucumber [48].

**Table 3:** Assay of combination of antagonist fungi and CNPs against *F. equiseti*

pathogen	[CNPs], (mg/ml)	Inhibition rate (%)		
		*T. longibrachiatum*	*P. polonicum* st.1	*P. polonicum* st.2
*F. equiseti* st.1	0	60.00	40.00	49.41
	0.172	65.88	42.35	60.00
*F. equiseti* st.2	0	62.74	51.41	58.82
	0.172	71.05	55.52	66.70

**Table 4:** Comparson between the antifungal effect of *Tr. longibrachiatum* combined with CNPs and the other reported antifungal against *F. equiseti*

No	Controlling agent	Inhibition rate (%)	References
1	*T. longibrachiatum* combined with CNPs (0.172 mg/ml)	71.05	Our work
2	*Azotobacter nigricans*	50	[19]
3	*Bacillus subtilis*	66	[20]
4	*Streptomyces bellus*	55	[16]
5	*Talaromyces* strain DYM25	71.46	[10]
6	*Streptomyces netropsis*	40	[49]
7	CuNPs (0.2 mg/ml)	51.28	[50]
8	CNPs (1 mg/ml)	56.5	[34]

In order to enhance the antifungal activity of *T. longibrachiatum, P. polonicum* st.1 and *P. polonicum* st.2 against the two strain *F. equiseti*, the antagonistic fungi were firstly cultivated in PDA media containing CNPs with a concentration of 0.172 mg/ml that achieves the maximum inhibition for both *F. equiseti* st.1 and *F. equiseti* st.2 to produce a combination of *T. longibrachiatum* with CNPs, *P. polonicum* st.1 with CNPs as well as *P. polonicum* st.2 with CNPs. After 7 days of incubation, the effect of these combinations against the two strain *F. equiseti* were studied using the dual plate method. The results in Table 3 shows that combination of CNPs with *T. longibrachiatum* increases its antifungal activity from 60% to 65.88% and from 62.74% to 71.05% against *F. equiseti* st.1 and *F. equiseti* st.2, respectively. Also, CNPs increases the antifungal activity of *P. polonicum* st.1 and *P. polonicum* st.2 from 40% to 42.35% and from 51.41% to 55.52% against *F. equiseti* st.1, respectively. In case of *F. equiseti* st.2, CNPs lead to increase the inhibition rate from 51.41% to 55.52% and from 58.82% to 66.7% using *P. polonicum* st.1 and *P. polonicum* st.2, respectively. Alike, it is stated that biogenic CNPs from four different fungal sources in combination with *Trichoderma asperellum* was effective in suppressing mycelial growth *Fusarium oxysporum* and other soil borne pathogenic fungi [38]. These results confirm the high biological control of CNPs against the two strain *F. equiseti.*

There are a few studies dealing with biocontrol of *F. equiseti* based on the use of microorganisms including bacteria, fungi and actinomycetes. Table 3 presents a comparison between the effect of *T. longibrachiatum* combined with CNPs, which achieve the maximum inhibition rate, and the other reported antifungal against *F. equiseti*. According to Table 3, both our combination of *T. longibrachiatum* with CNPs and *Talaromyces* strain DYM25 [10] exhibit the maximum inhibition rate (about 71%) against *F. equiseti.*

## Conclusion

In conclusion, the growth of tomato wilt pathogen, *F. equiseti* can be controlled using CNPs, *T. longibrachiatum, P. polonicum* st.1 and *P. polonicum* st.2 with high inhibition rate. The use of CNPs is ecofriendly, biodegradable and anti- *F. equiseti* with higher inhibition rate compared with antagonistic fungi. Moreover, the combination of CNPs with *T. longibrachiatum* or *P. polonicum* strains enhance their antifungal activity against *F. equiseti.*

## Data Availability

DNA sequences for the isolated fungi are available in GenBank with accession numbers ON533654, ON533655, ON533656, ON533657, ON533658. All the datasets analysed during the current study are available from the corresponding author on reasonable request.
